# Toward Development of Neuron Specific Transduction After Systemic Delivery of Viral Vectors

**DOI:** 10.3389/fneur.2021.685802

**Published:** 2021-08-26

**Authors:** Dylan J. Finneran, Ikenna P. Njoku, Diego Flores-Pazarin, Meghana R. Ranabothu, Kevin R. Nash, David Morgan, Marcia N. Gordon

**Affiliations:** ^1^Translational Neuroscience, College of Human Medicine, Michigan State University, Grand Rapids, MI, United States; ^2^Department of Molecular Pharmacology and Physiology, Morsani College of Medicine, University of South Florida, Tampa, FL, United States

**Keywords:** adeno-associated viral vectors, AAV, CaMKIIα promoter, Syn1 promoter, intravenous delivery

## Abstract

Widespread transduction of the CNS with a single, non-invasive systemic injection of adeno-associated virus is now possible due to the creation of blood-brain barrier-permeable capsids. However, as these capsids are mutants of AAV9, they do not have specific neuronal tropism. Therefore, it is necessary to use genetic tools to restrict expression of the transgene to neuronal tissues. Here we compare the strength and specificity of two neuron-specific promoters, human synapsin 1 and mouse calmodulin/calcium dependent kinase II, to the ubiquitous CAG promoter. Administration of a high titer of virus is necessary for widespread CNS transduction. We observed the neuron-specific promoters drive comparable overall expression in the brain to the CAG promoter. Furthermore, the neuron-specific promoters confer significantly less transgene expression in peripheral tissues compared with the CAG promoter. Future experiments will utilize these delivery platforms to over-express the Alzheimer-associated pathological proteins amyloid-beta and tau to create mouse models without transgenesis.

## Introduction

Adeno-associated virus (AAV), a single-stranded DNA virus, is a member of the Dependovirus subfamily of the Parvoviridae family of viruses (1, 2). AAV has been used extensively in pre-clinical studies and clinical trials due to its efficient transduction of non-dividing cells, long-lasting expression, and low immunogenicity. Many factors may influence the transduction efficiency and targeting of AAV gene therapy such as route of delivery, use of a self-complementary AAV genome, and serotype (3). Most preclinical experiments and clinical trials focusing on neurodegeneration deliver AAV gene therapy by intracranial injection into the brain (4, 5). In addition to the need for a craniotomy, only a portion of the brain is transduced and there is a gradient of transduction diminishing with distance from the site of injection. However, neurodegenerative diseases afflict multiple brain regions. A major challenge is distributing gene therapy to the large and spatially dispersed volumes of brain affected. Moreover, it would be less invasive to administer AAV intravenously. Recently, capsid variants of AAV9 have been engineered using directed evolution that are more efficient at crossing the blood-brain barrier than other serotypes (6, 7). Capsid variants named PHP.B or PHP.eB gain entry into the CNS of C57BL/6J mice by binding to specific isoforms of endothelial Ly6a receptor (8), allowing for broader and more uniform transduction of the CNS than intraparenchymal injections.

As the PHP.B family of capsids are mutants of AAV9, they share AAV9's tropism and transduce primarily neurons within the CNS (3, 9). Consequently, the PHP.B family of capsids have been used to test therapeutics in mouse models of both Parkinson's disease (10) and Rett syndrome (11), proving they are a useful tool for preclinical research. However, AAV9 also transduces peripheral tissues after intravenous delivery, especially liver, heart, and skeletal muscle (12). For optimal therapeutic effectiveness in the CNS, it is necessary to restrict expression of the transgene to neuronal tissue in order to avoid off-target effects of the transgene. Work with intraparenchymal injections of varying AAV serotypes has identified the neuron-specific promoters human synapsin1 (hSyn1) and calcium/calmodulin dependent protein kinase IIA (CaMKIIα) as reliable, robust promoters that restrict expression to neurons (13, 14). However, a comparison has not been made after intravenous administration of PHP.eB in mice.

Here, we chose to focus on systemic delivery of the blood-brain barrier-permeable PHP.eB AAV capsid expressing green fluorescent protein (GFP). We examined the transduction efficiency and strength of transgene expression driven by three different promoters in both the brain and select peripheral tissues. We evaluated the relative strength of the neuron selective hSyn1 and CaMKIIα promoters and assess their respective abilities to restrict peripheral expression, as compared to the ubiquitous CAG promoter, after intravenous administration of the PHP.eB variant of AAV. Animals were injected in the lateral tail vein with PHP.eB-GFP driven by the CAG promoter, hSyn1 promoter, or CaMKIIα promoter. We determined the three promoters yield roughly the same overall expression in brain, with slight differences between brain regions. As expected, the neuron-specific promoters successfully minimized GFP expression in the peripheral tissues, particularly in the liver. The strong neuronal expression in the CNS, with little expression in peripheral tissues, make either of these neuron-specific promoters a good choice for preclinical gene therapeutic testing in neurodegenerative disease.

## Materials and Methods

### Cloning & Adeno-Associated Virus Production

The expression vector pTR-GFPW was generated by subcloning GFP into the vector pTR12.1 MCS (15) along with the woodchuck hepatitis virus posttranscriptional regulatory element (WPRE) into the Age I/Nhe I and Cla I restriction sites, respectively. The cloning vectors pTR-Syn-MCSW and pTR-CaMK-MCSW were generated to have the human synapsin promoter or mouse CaMKIIα promoter and WPRE for strong gene expression (16). Human synapsin promoter was cloned using polymerase chain reaction with the following primers GAGGGTACCGAGGGCCCTGCGTATGAGTGC and GAGAAGCTTCTCGACTGCGCTCTCAGGC and cloned using Kpn I and Hind III restriction enzymes of the pTR12.1 MCSW vector. Promoter sequence was confirmed by DNA sequencing (Genbank M55301.1). Mouse CaMKIIα promoter sequence was isolated from mouse genomic DNA using PCR with the following primers: ATTAGAATTCCATTATGGCCTTAGGTCACTTCATCTCC and ATTAAAGCTTGCTGCCCCCAGAACTAGGG. Promoter sequence was confirmed by DNA sequencing (RefSeq NC_000084.7). See Supplementary Table 1 for promoter sequences. These elements were flanked by AAV2 terminal repeats and the BGH poly A signal for transcription termination. pTR-Syn-GFPW and pTR-CaMK-GFPW were generated by subcloning GFP into the Age I and Nhe I cloning sites of the respective cloning vectors. The resulting clones were screened for presence of terminal repeats by Sma I digest and expression by transfection into HT22 cells. The construct encoding PHP.eB (pUCmini-iCAP-PHP.eB) was a generous gift from Viviana Gradinaru (7) (plasmid #103005; http://n2t.net/addgene:103005; RRID: Addgene_103005) with a biological material transfer agreement. Recombinant AAV-PHP.eB particles were generated using the triple transfection method as described previously (17) and quantified using the dot-blot method with a non-radioactive biotinylated probe for GFP generated by PCR (18).

### Mice and Breeding

All animal experiments were conducted in accordance with the National Research Council “Guide for the Care and Use of Laboratory Animals” and were approved by the Institutional Animal Care and Use Committee at Michigan State University. Study animals were 6–12-month-old C57BL/6 mice maintained on a 12-h light/dark cycle. The mice were given food and water *ad libitum*.

### AAV Injection and Tissue Collection

For the initial dose-response study, mice were restrained and injected in the lateral tail vein with 100 μL of PHP.eB-CAG-GFP at 5 × 10^13^, 5 × 10^12^, 5 × 10^11^, and 5 × 10^10^ viral genomes (vg) per mL diluted in sterile PBS using a 30-gauge needle. Final doses were thus 5 × 10^12^, 5 × 10^11^, 5 × 10^10^ and 5 × 10^9^ vg per mouse (*n* = 3 mice for 5 × 10^9^ vg; *n* = 4 mice for the remaining groups). Control mice were injected with 100 μL of sterile PBS (*n* = 2 mice). For the comparison of the ubiquitous CAG promoter with the neuron-specific promoters, mice were injected in the lateral tail vein with 100 μL of 5 × 10^13^ vg/mL of PHP.eB-CaMKIIα-GFP, PHP.eB-hSyn1-GFP or PHP.eB-CAG-GFP (*n* = 4 mice per group).

Four weeks after injection, mice were weighed and injected with a euthanizing solution containing pentobarbital (100 mg/kg) and phenytoin (12.5 mg/kg). The deeply anesthetized mice were transcardially perfused with 25 mL of 0.9% saline. Brain, liver, lung, heart, kidney, and skeletal muscle (gastrocnemius) were collected immediately after perfusion. Peripheral tissues were snap frozen on dry ice. The right hemisphere of the brain was dissected on ice into four regions, which were then frozen on dry ice for biochemical analysis. The left hemisphere was fixed in 4% paraformaldehyde for 24 h at 4 °C. The fixed hemisphere was cryoprotected in sucrose by successive 24-h incubations in 10, 20, and finally 30% sucrose (mass/vol). Brains were frozen on a cold stage and 25 μm thick horizontal sections were collected with a sliding microtome. Sections were stored in PBS with 10 mM sodium azide at 4 °C.

### Tissue Homogenization and Western Blotting

Liver, lungs, muscle, heart, and kidney were pulverized with a mortar and pestle on dry ice. The fine particles were then weighed, thawed on ice, and lysed in TBS with protease inhibitor cocktail (Sigma Aldrich, St. Louis, MO, USA, Cat. No. P8340), and Benzonase (Sigma Aldrich, St. Louis, MO, USA, Cat. No. e1014-25ku; 25 U/mL final concentration) at 10 vol/wt of tissue. The homogenate lysate was then sonicated (3 × 10 s) and centrifuged at 1,000 × g for 1 min at 4 °C. The resultant supernatant was then collected into a new labeled Eppendorf tube and stored for downstream protein extraction.

Brain regions (cerebellum, hippocampus, anterior, and posterior cortex) were homogenized in Qiagen buffer RLT + β-mercaptoethanol (1:100) using a battery-operated Argos micro-grinder (RPI, Mount Prospect, IL, USA, Cat. No. 299220). DNA, RNA, and protein were extracted simultaneously from the brain samples using the Qiagen AllPrep Kit per the manufacturer's protocol (Qiagen USA, Cat. No. 80004). Total protein concentration was determined using Pierce BCA protein assay (ThermoFisher Scientific, Waltham, MA, USA), after which 10 μg of total protein was used per sample for western blotting. Anti-GFP antibody (Abcam, Cambridge, UK; Cat. No. ab13970) was used to determine the expression of the reporter dye. Samples were run on pre-cast 4–20% TGX Stain-Free gels (Bio-Rad, Hercules, CA, USA). The total-protein dye was activated by UV irradiation prior to transfer to a nitrocellulose blot and total protein transferred to the blot was measured. The blots were then blocked in 5% milk in TBS and probed with primary antibody diluted in 5% milk in TBST overnight at 4 °C. Blots were washed in TBST and incubated in secondary antibody diluted in TBST 2 h at room temperature. Western blots were imaged on Bio-Rad ChemiDoc MP Imaging System and analyzed using Bio-Rad Image Lab Software for PC Version 6.0. All signals were normalized to the total protein signal acquired after transfer to nitrocellulose.

### RNA Quantification and Real-Time PCR

RNA samples were reverse transcribed to cDNA using Bio-Rad Advanced iScript RT (Cat. No. 1725038) and the relative expression of the transgene was determined using quantitative PCR. Primers for the target genes (WPRE: Fwd – GGCTGTTGGGCACTGACAAT; Rev - CCGAAGGGACGTAGCAGAAG) and the housekeeping gene (18S rRNA: Fwd – GTAACCCGTTGAACCCCATT; Rev - CCATCCAATCGGTAGTAGCG) were used per the manufacturer's recommended protocol. Genomic DNA was assayed for the WPRE and the mouse glucagon gene was used as the single copy gene for normalization (Bio-Rad Cat. No. qMmuCED0044332). Twenty nanograms of starting material was loaded per well and threshold fluorescence cycle was measured on a Bio-Rad CFX96 thermal cycler (95 °C for 10 min, followed by 40 cycles of: 95 °C for 15 s, 60 °C for 1 min). Quantitative PCR data was analyzed using the relative standard curve method and normalized to the single copy gene (19).

### Immunohistochemistry

Six to eight sections spanning the brain were chosen for analysis. Immunohistochemical (fluorescence and chromogenic) experiments were performed as described previously (20). Floating sections for each animal were placed into a multi-sample staining tray.

For the chromogenic experiments, endogenous peroxidases were blocked (10% methanol, 3% hydrogen peroxide in PBS for 15 min) and tissue was permeabilized (0.2% lysine, 0.1% Triton X-100 in PBS for 30 min). Sections were incubated overnight in the appropriate primary antibody: anti-GFP (Abcam, Cambridge, UK; Cat. No. ab13970; at 1:30,000). Sections were washed three times in PBS, then incubated for 2 h with corresponding biotinylated secondary antibody (Vector Laboratories, Burlingame, CA, USA; Cat. No. BA-9010-1.5; at 1:3,000). The tissue was again washed and incubated with Vectastain Elite ABC Kit (Vector Laboratories) for enzyme conjugation. Finally, sections were stained using 0.05% diaminobenzidine and 0.03% hydrogen peroxide for 5 min. Each immunohistochemical assay omitted some sections from primary antibody incubation to evaluate nonspecific binding of the secondary. Sections were mounted onto slides, dehydrated, and cover slipped.

Fluorescence staining was performed as above but omitted the blocking of endogenous peroxidases. The primary antibody concentration used were anti-GFP (1:3,000), anti-GFAP (Agilent Technologies, Santa Clara, CA, USA; Cat. No. Z033401-2; at 1:3,000), and anti-NeuN (EMD Millipore, Darmstadt, Germany; Cat. No. MAB377B; at 1:300). Sections were washed three times in PBS, then incubated for 2 h with corresponding secondary antibodies: anti-chicken-Alexa 488 (Thermo Fisher Scientific, Waltham, MA, USA; Cat. No. A32931: at 1:3,000), anti-rabbit-Alexa 647 (Thermo Fisher Scientific, Waltham, MA, USA; Cat. No. A32733: at 1:3,000), and streptavidin-Alexa 594 (Thermo Scientific, Waltham, MA, USA; Cat. No. S32356: at 1:3,000). The tissue was washed again, the sections were mounted onto slides, and cover slipped with ProLong Glass with NucBlue (ThermoFisher Scientific, Waltham, MA, USA; Cat. No. P36981).

For the chromogenic stains, the area of positive staining across the entire brain section, the hippocampal formation, the cortex, and cerebellum was analyzed. Stained sections were imaged using a Zeiss Axioscan.Z1 scanning microscope. Neurocyte IAE software (created by Andrew Lesniak) used hue, saturation, and intensity (HSI) to segment the images and these values were held constant for analysis of every section of every animal in each stain. These HSI values were established on sections with high and low levels of staining to reliably segment positive staining over background. The percent area staining was determined mathematically by dividing the number of pixels above threshold by the total number of pixels in the region of interest (20). Fluorescent images were taken on a Nikon A1+ scanning laser confocal microscope. Three 20 × fields of view were taken per brain region across three to four sections per animal. A mask of fluorescence intensity above threshold was created in iMARIS software (Oxford Instruments, Abingdon, UK) and the seed-growing algorithm was used to detect individual NeuN-positive and GFP-positive cells, respectively. The number of double-positive cells was determined by the intersection of these two sets and manually checked for accuracy. Percentage of GFP+NeuN+ neurons cells was mathematically determined by dividing the number of double positive cells by the number of NeuN+ cells.

### Statistical Analysis

Statistical analysis was performed using GraphPad Prism Statistics Software version 9.0.1 (San Diego, CA, USA). A one-way ANOVA with Tukey's *post-hoc* was performed to analyze IHC data whereas a two-way ANOVA with Tukey's *post-hoc* was used to analyze western and qPCR data. The threshold for significance was set at *p* < 0.05.

## Results

### A High Dose of PHP.eB Is Required for Robust, Widespread CNS Transduction

**Figure 1:**
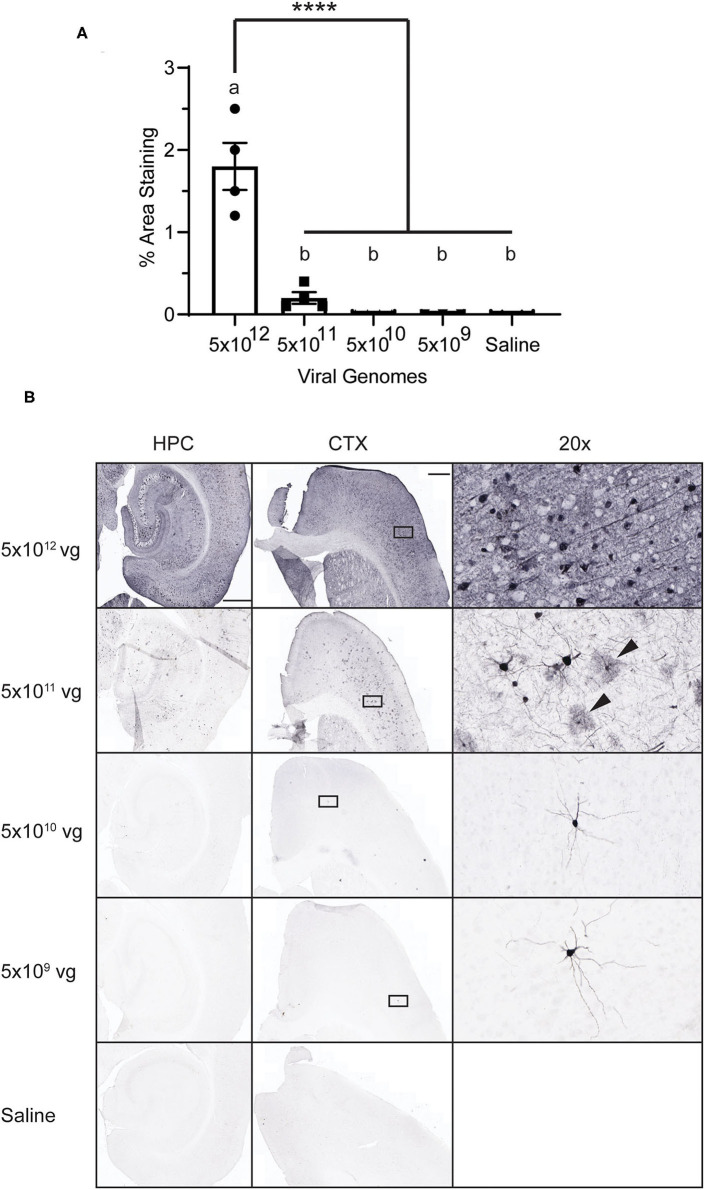
AAV Dose-Response. Mice were injected with PHP.eB-CAG-GFP or saline and tissue was collected 4 weeks later. **(A)** Graph of percent area staining of GFP. Data points represent values for each mouse; mean ± SEM (*n* = 3 for 5 × 10^9^ vg; *n* = 4 for remaining groups) are indicated. **** denotes *p* < 0.0001 by one-way ANOVA and Tukey's *post-hoc* test. Bars annotated with the same lower-case letter are not statistically different from each other. **(B)** Representative images with anti-GFP staining. Rectangles on the CTX panels indicate area enlarged on the right (20 × panels). Arrowheads indicate presumptive glial cells. CTX and HPC scale bar = 500 μm, 20 × scale bar = 50 μm. vg, viral genomes; CTX, cortex; HPC, hippocampus.

PHP.eB is an engineered variant of AAV9 that more efficiently crosses the blood-brain barrier to infect the CNS (7). First, we sought to determine the optimal titer of PHP.eB-CAG-GFP to deliver intravenously via the lateral tail vein that would induce robust and widespread expression of GFP in the CNS. We administered four titers of PHP.eB-CAG-GFP and assessed GFP expression 4 weeks after injection. We observed that while cells positive for GFP were observable at all titers of virus, only the highest titer tested, 5 × 10^12^ vg per mouse, induced robust GFP expression (Figures 1A,B). As previously reported (7, 21), we also observed broad transduction throughout the brain, including the cortex, hippocampus, striatum, and cerebellum. We also observed that while many of the cells transduced had neuronal morphology, there were some presumptive astrocytes transduced as well (Figure 1B arrowheads), consistent with other reports (7). For our comparison with neuron-specific promoters, we chose to inject the highest titer tested.

### The CaMKIIα Promoter Drives Greater Expression Than hSyn1 at the RNA Level

**Figure 2:**
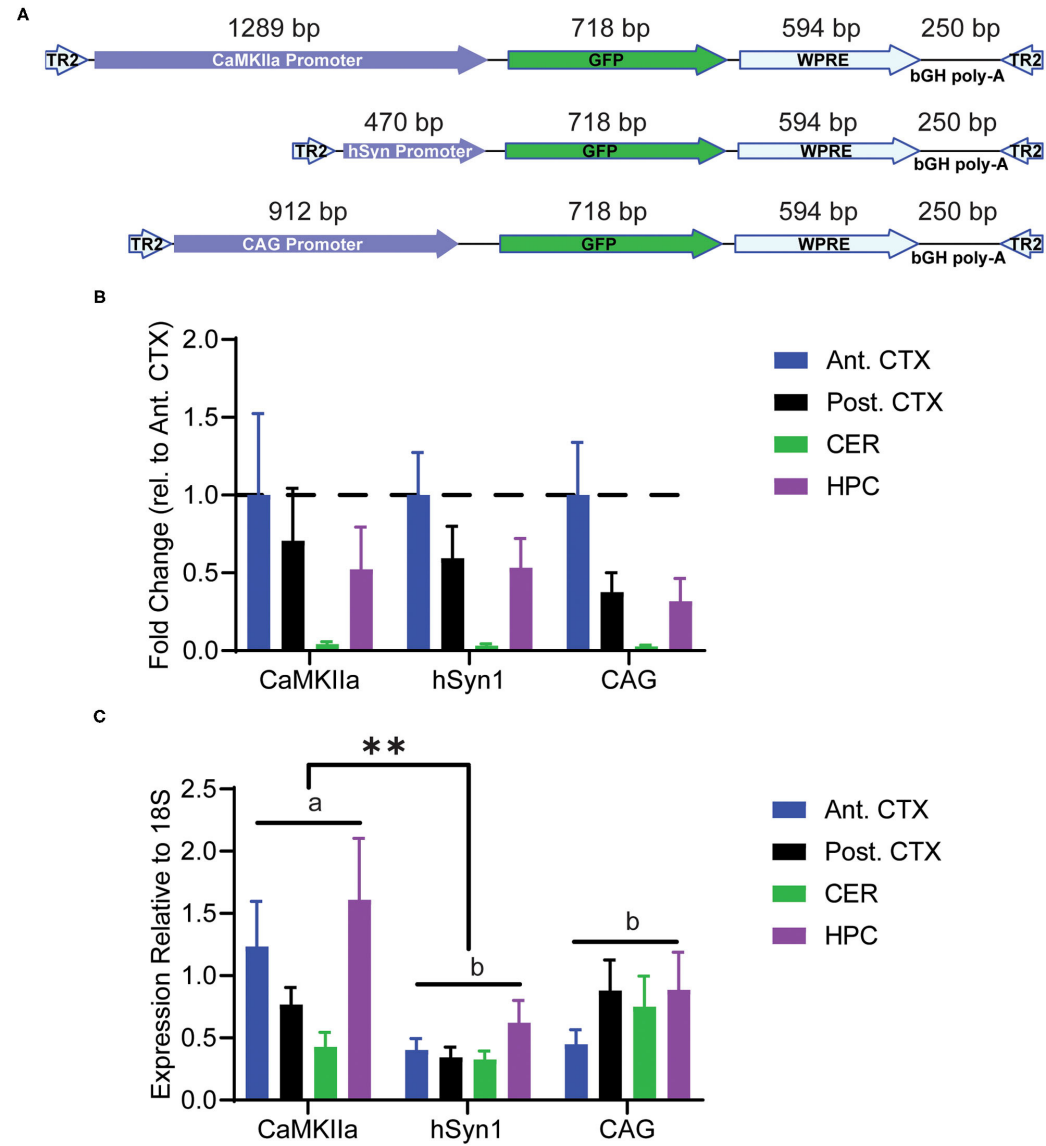
Transduction of discrete brain regions. Mice were injected with PHP.eB-GFP driven by either the CaMKIIα, hSyn1, or CAG promoters and tissue was collected 4 weeks later. **(A)** Schematic of the AAV constructs. **(B)** Graph of viral genomes per mouse genome. A main effect of brain region was observed by two-way ANOVA. **(C)** Graph of GFP RNA expression relative to 18S rRNA assayed by RT-qPCR. Data are presented as mean ± SEM (*n* = 4). A main effect of promoter was observed with CaMKIIα driving significantly greater overall expression than hSyn1. **denotes *p* < 0.001 by two-way ANOVA and Tukey's *post-hoc* test. Bars annotated with the same lower-case letter are not statistically significant from each other. CaMKIIα, calmodulin dependent protein kinase IIα; hSyn1, human synapsin 1; Ant. CTX, anterior cortex; Post. CTX, posterior cortex; CER, cerebellum; HPC, hippocampus.

In order to specifically target neurons, we chose to investigate GFP expression driven by the specific promoters human synapsin 1 or mouse CaMKIIα, as well as the ubiquitous CAG promoter (Figure 2A). Animals were administered PHP.eB-GFP intravenously in the tail vein and tissue was collected 4 weeks after injection. The viral genomes per mouse genome were determined by qPCR of genomic DNA. We observed the same pattern between promoters of viral genome load (Figure 2B). The tropism of the PHP.eB virus was not influenced by the promoter with highest transduction efficiency in anterior cortex and lowest in cerebellum. Expression of the transgene was assessed by qRT-PCR in the anterior cortex, posterior cortex, hippocampus, and cerebellum. We observed a significant main effect of promoter by ANOVA with the CaMKIIα promoter driving greater overall RNA transcription than the hSyn1 promoter (Tukey's test *p* < 0.01). However, there was no statistical difference between CaMKIIα expression and CAG expression (Tukey's test; Figure 2C). Although GFP RNA expression appeared highest in hippocampus and anterior cortex after injection of PHP.eB-CaMKIIα-GFP, there was no main effect of brain region by two-way ANOVA.

### Brain Region-Specific Differences in Transgene Expression at the Protein Level

**Figure 3:**
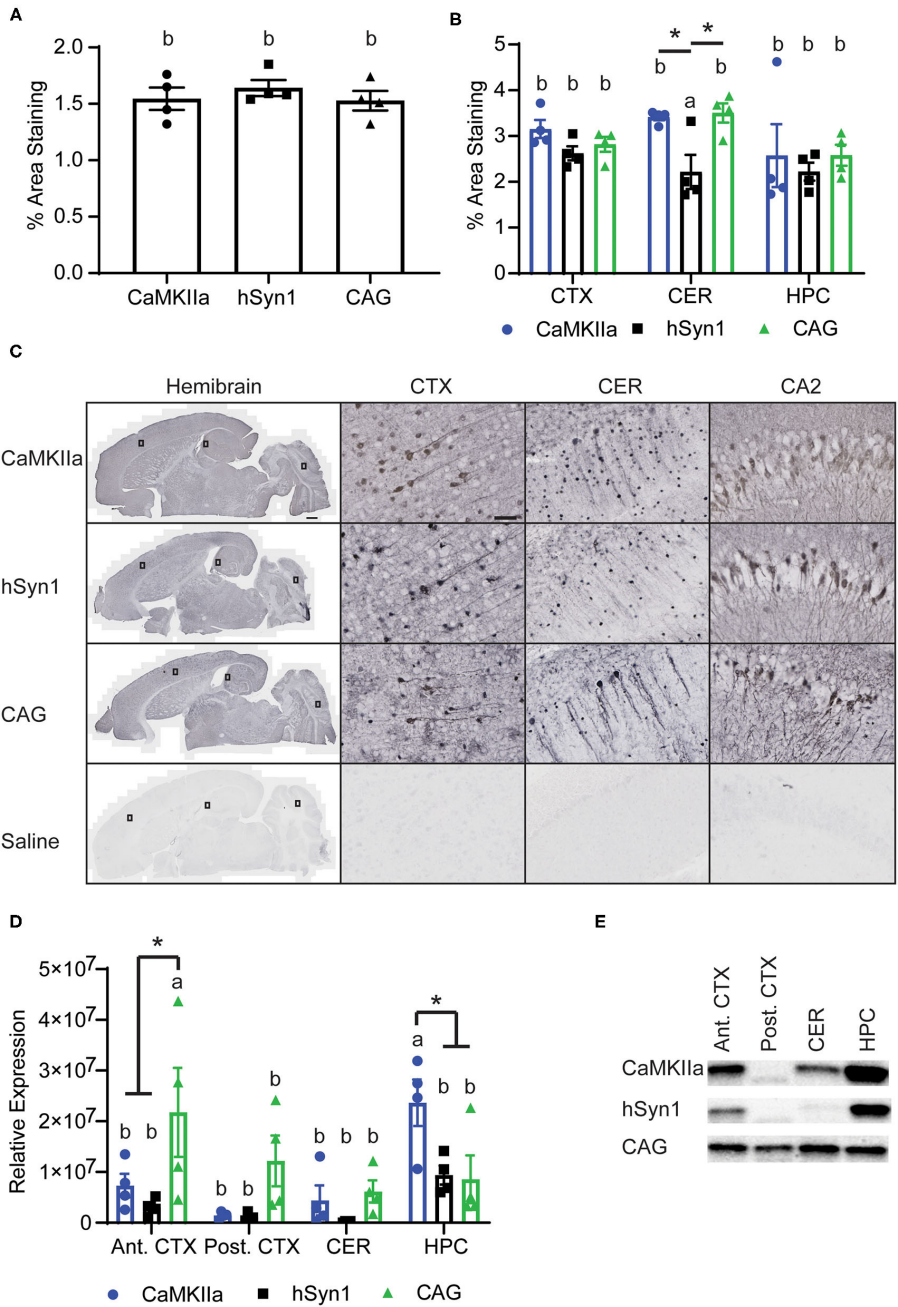
CNS Expression of GFP protein. **(A)** Graph of percent area staining of GFP from hemibrain sections. **(B)** Graph of percent area staining of GFP in the cortex, cerebellum, and hippocampus. *denotes *p* < 0.05 by one-way ANOVA and Tukey's *post-hoc* test. **(C)** Representative images of anti-GFP staining. Small rectangles on the hemibrain panels display the area enlarged in the panels to the right. Scale bars represent 50 μm in CTX, CA2, and CER panels, and 500 μm in hemibrain panel. **(D)** Graph of GFP expression in discrete brain regions by western blot. GFP signals were normalized to total protein per lane using a total protein stain. The CAG promoter drove the greatest expression in the anterior cortex whereas the CaMKIIα promoter drove the greatest expression in the hippocampus. *denotes *p* < 0.05 by two-way ANOVA and Tukey's *post-hoc* test. Data points represent values for each mouse; mean ± SEM (*n* = 4) are indicated. Bars annotated with the same lower-case letter are not statistically significant from each other. **(E)** Representative images of Western blotting. CaMKIIα, calmodulin dependent protein kinase IIα; hSyn1, human synapsin 1; Ant. CTX, anterior cortex; Post. CTX, posterior cortex; CER, cerebellum; HPC, hippocampus; CA2, subfield of hippocampus.

We assessed transgene expression on the protein level by IHC. We did not observe a significant difference in GFP staining when analyzed over the entire section, with all three promoters driving comparable levels of GFP immunoreactivity throughout the brain (Figures 3A,C). As expected, we did not observe any cells expressing GFP with glial morphology in animals injected with either hSyn1-GFP or CaMKIIα-GFP (Figure 3C). Examining GFP expression within specific brain regions by IHC and western blot, we did observe significant differences between promoters. As measured by IHC, the hSyn1 promoter drove significantly less GFP expression in the cerebellum than either the CaMKIIα or CAG promoters (Figure 3B). By western blot, the CaMKIIα promoter drove the highest expression in the hippocampus whereas the CAG promoter drove the highest expression in the anterior cortex (Figures 3D,E).

**Figure 4:**
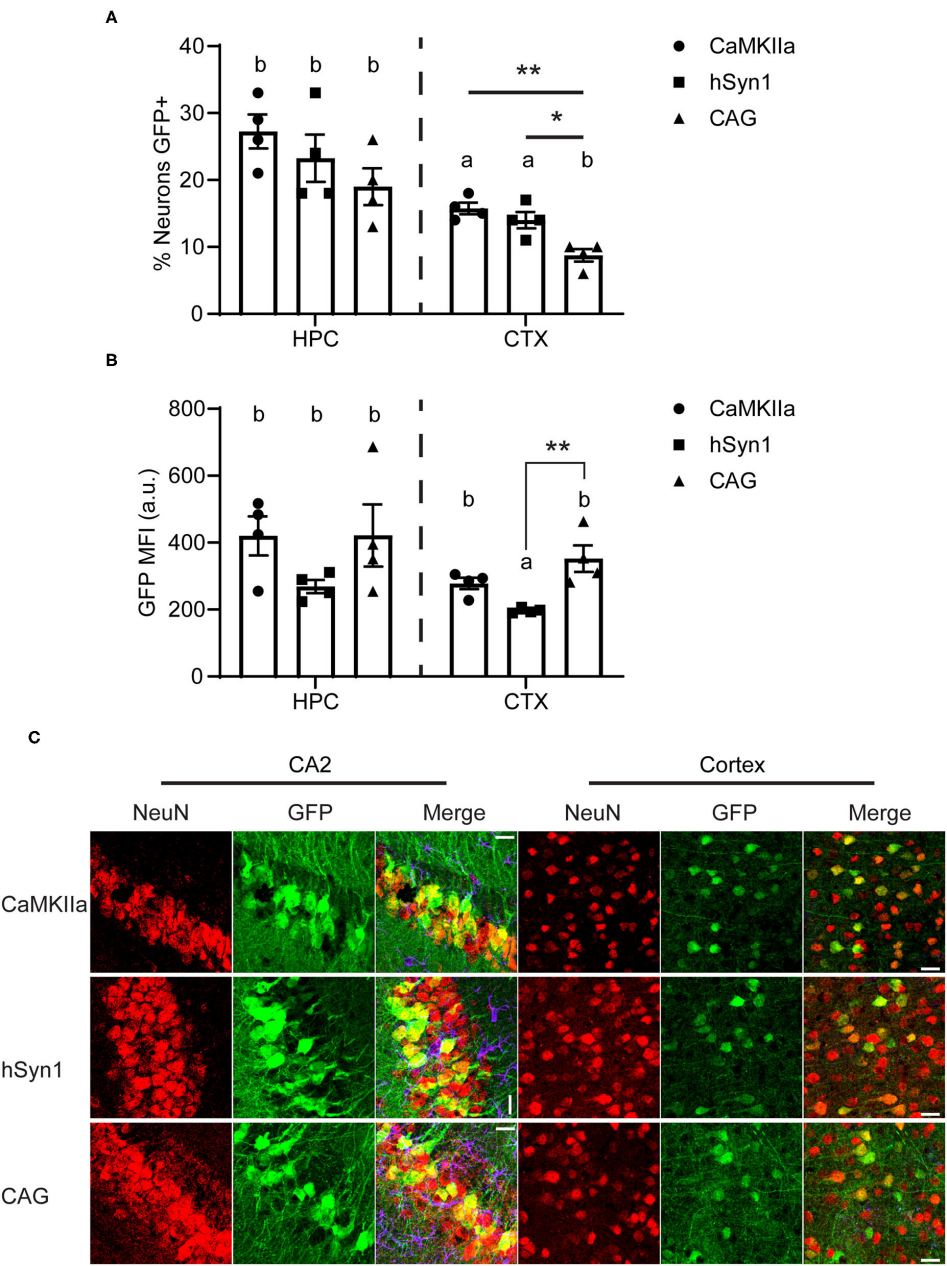
The neuron-specific promoters transduce a greater percentage of NeuN+ cells than the CAG promoter. **(A)** Graph of approximate percentage of NeuN-positive cells also expressing GFP. Both neuron-specific promoters transduced significantly more cells in the cortex than the ubiquitous CAG promoter. **(B)** Graph of mean fluorescent intensity of GFP signal from GFP+NeuN+ cells. The CAG promoter drove greater per-neuron expression of GFP in the cortex than the hSyn1 promoter. Data points represent values for each mouse; mean ± SEM (*n* = 4) are indicated. ** denotes *p* < 0.01; * denotes *p* < 0.05 by one-way ANOVA and Tukey's *post-hoc* test. Bars annotated with the same lower-case letter are not statistically significant from each other. **(C)** Representative micrographs of the hippocampus and cortex. Apparent rotations in CA2 orientation resulted from section placement on slides. Similar areas indicated with rectangles on Figure 3C are enlarged here. Scale bar represents 25 μm for all panels. CaMKIIα, calmodulin dependent protein kinase IIα; hSyn1, human synapsin 1; Ant. CTX, anterior cortex; Post. CTX, posterior cortex; CER, cerebellum; HPC, hippocampus; CA2, subfield of hippocampus.

We next estimated the neuronal transduction efficiency for each construct by analyzing NeuN staining. All promoters transduced ~25% of the NeuN+ pyramidal neurons in the hippocampus and there was no statistical difference between promoters in the percentage of NeuN+ neurons transduced. Regardless of promoter, we observed that a greater percentage of NeuN+ cells were transduced in the hippocampus than in the cortex. Approximately 15% of NeuN+ cortical neurons were transduced using either CaMKIIα or hSyn1 promoters, while significantly fewer neurons were transduced using the CAG promoter (Figures 4A,C). This contrasts with our western data showing strong GFP protein signals in cortex using the CAG promoter (Figure 3C). However, examining the mean fluorescent intensity of GFP expression in NeuN+GFP+ cells, we observed significantly greater fluorescence intensity in the cortex of animals that received CAG-GFP compared to hSyn1-GFP (Figures 4B,C). This may account for the discrepancy between the expression data by western (Figure 3D) and the percentage of neurons transduced (Figure 4A).

### Both Neuron-Specific Promoters Prevent Peripheral Expression of GFP

**Figure 5:**
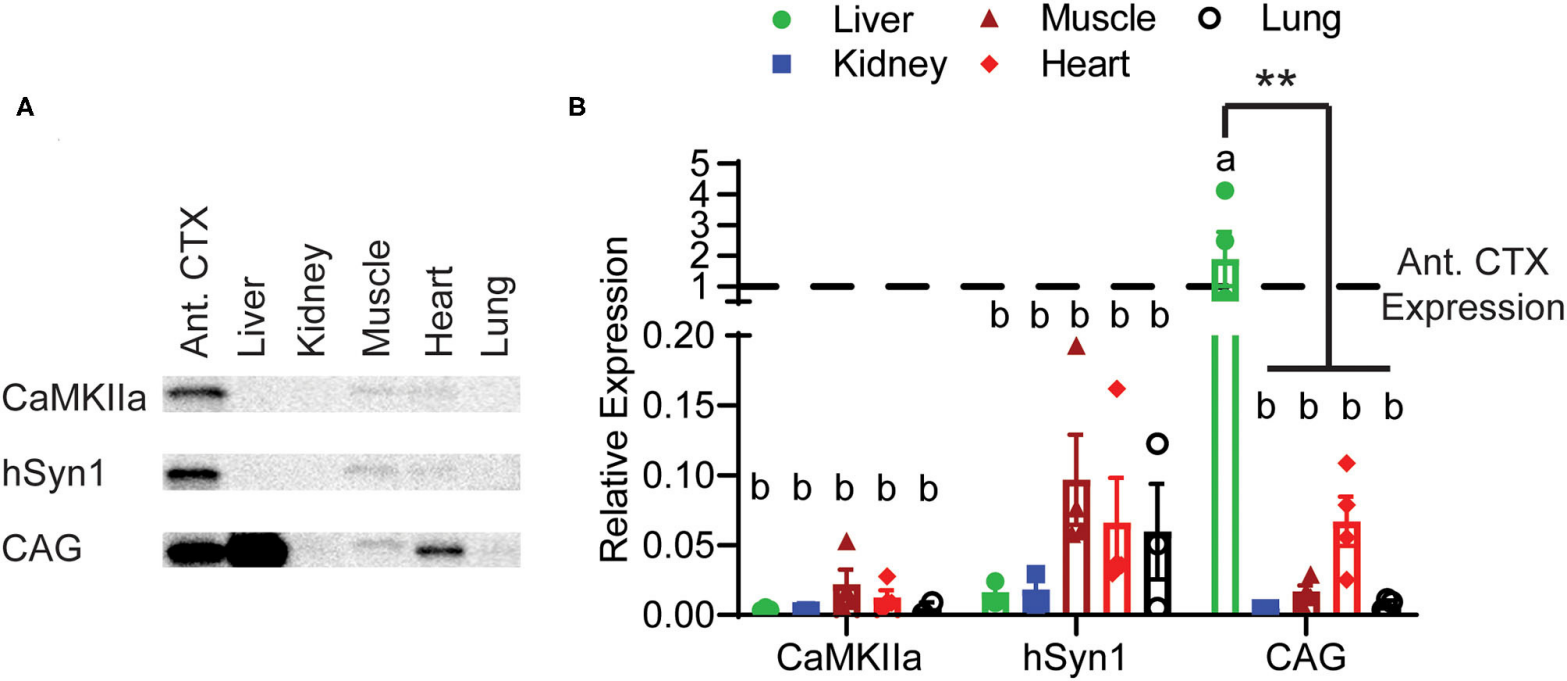
Peripheral expression of GFP limited by neuron-specific promoters. **(A)** Representative images of Western blotting. **(B)** Expression of GFP in peripheral tissues relative to expression in the anterior cortex. Both the hSyn1 and CaMKIIα promoters drove significantly less GFP expression in the liver than the CAG promoter. Dashed line indicates level of expression in anterior cortex. Data points represent values for each mouse; mean ± SEM (*n* = 4) are indicated. **denotes *p* < 0.05 by two-way ANOVA and Tukey's *post-hoc* test. Bars annotated with the same lower-case letter are not statistically significant from each other.

Finally, we assessed GFP expression driven by these three constructs in peripheral tissues. Perhaps unsurprisingly due to it being a highly vascularized organ, we observed approximately twice the GFP expression in liver than the anterior cortex in animals injected with CAG-GFP. In the other tissues examined, there was very low GFP expression even with the CAG promoter. Both neuron-specific promoters had almost undetectable levels of GFP expression in any peripheral tissue. Expression in the liver was significantly lower using the CaMKIIα and hSyn1 promoters compared with the CAG promoter (Figures 5A,B). The CaMKIIα promoter seemed to express less than the hSyn1 promoter in muscle, heart, and lung.

## Discussion

We determined the titer of virus necessary for robust CNS expression after intravenous administration of PHP.eB-GFP. We also demonstrated the CNS-selective expression of GFP driven by the mouse CaMKIIα and human synapsin 1 promoters as compared to the ubiquitous CAG promoter. Interestingly, although the CAG promoter is known to drive high levels of expression, the two neuron-specific promoters drove GFP expression in brain regions to levels comparable to the CAG promoter. We observed some differences in promoter strength when comparing specific brain regions, so different experiments might find greater utility with one promoter over the other. The greatest advantage of these neuron-specific promoters is the ability to significantly diminish expression of the transgene in peripheral tissues.

In accordance with prior reports, we observed that a large titer of virus is required for robust CNS expression with transduction efficiency rapidly declining with smaller doses administered (21). Based on morphology, the CAG-GFP construct transduced both glial and neuronal cells. Interestingly, we observed more cells with glial morphology at the second-highest titer, although this may be due to greater visibility with less widespread GFP expression as opposed to differences in transduction (6). We did not observe any glial expression in animals injected with the neuron-specific promoters. Similar to (3), we observed a high transduction efficiency specifically in the CA2 region of the hippocampus with all three promoters. This can be seen in others' work as well (3, 22, 23) and likely results from greater multiplicity of infection due to viral tropism for these neurons rather than enhanced promoter expression.

Prior studies have compared the relative strength of the hSyn1 and CaMKIIα promoters and found they drive comparable levels of expression (24). We found significantly greater RNA levels driven by CaMKIIα but comparable levels of protein by western blot. Both promoters also transduced approximately the same proportion of NeuN-positive neurons in the cortex and hippocampus. Interestingly, the virus with the CaMKIIα promoter transduced a significantly greater number of neurons than the CAG promoter in the cortex. However, we observed significantly greater GFP expression per neuron in the cortex of animals that received CAG-GFP (Figure 4B). Non-neuronal GFP expression driven by the CAG promoter also likely contributed to the overall GFP expression measured by western blot (Figure 3D) while transducing fewer neurons (Figure 4A). The number of cells transduced is determined by the viral tropism which should be the same for all three promoters, so lower number of neurons expressing CAG-GFP suggests that there may be some regulation of the CAG promoter in specific neuron types. This would require further investigation exploring markers for different neuron subtypes.

We also observed a smaller percentage of NeuN+ cells were transduced by these viruses (up to 30%) than reported by (7) (up to 70%). This discrepancy may be attributed to two factors. First, the viral particles were generated at different institutions and tittered by different methods (Southern dot blot vs. qPCR). Secondly (7), administered their viruses into the retro-orbital sinus whereas we injected into the lateral tail vein. Administration of AAV9 via the retro-orbital sinus has been shown to yield greater CNS transduction than tail vein administration, presumably due to drainage from the orbital sinus into the cavernous sinus (25). We chose to inject in the lateral tail vein due to its translational relevance and a desire to preserve the animal's potential to perform in behavioral tasks requiring vision when this technique is applied to other disease modifying genes.

Both neuronal promoters produced significantly less expression of GFP in the liver, with trace to undetectable levels observed in the other peripheral tissues tested, compared with the CAG promoter. It should be noted that a shorter CaMKIIα promoter has been shown to drive similar levels of expression as the longer, 1.3 kb promoter tested here (24). This shorter promoter may be advantageous when expressing longer transgenes, given the relatively limited capacity of the AAV genome of 4.7 kb (26).

We chose the CaMKIIα promoter for study because it is expressed in forebrain regions at risk of developing pathology in Alzheimer's disease. An activator mouse expressing tetracycline-transactivator protein (tTa) under control of the CaMKIIα promoter has been used in transgenic mice to restrict expression of tetracycline responsive element-regulated transgenes to neurons in forebrain structures (27–29). This strain is used to regulate tau overexpression in the rTg4510 mouse model (29, 30). However, the phenotype of this mouse is influenced by the insertion site of the transgene (31). Future studies using AAV driven tau overexpression under the control of the CaMKIIα promoter might help to elucidate tau specific effects. In contrast, the Syn1 promoter may have a broader distribution in neurons across brain regions, making it a more useful construct depending on the experimental hypothesis to be addressed. However, in the regions we examined, the CaMKIIα-GFP construct had a similar rank order distribution throughout the brain as did the hSyn1-GFP construct (Figure 3D). Both neuron-specific promoters have been reported to drive expression in both excitatory and inhibitory neurons in the brain (13, 14). However, Nathanson et al. (13) observed a shift toward expression in inhibitory neurons as the titer of hSyn1-GFP virus decreased.

The promoters tested here may be combined with other AAV technologies to adapt to the specific needs of investigators. Here we included the WPRE enhancer to increase transgene expression. There are two conflicting reports indicating WPRE enhances (32) or does not enhance (33) gene expression driven by the CAG promoter. However, it has been reported to enhance gene expression driven by both the hSyn1 promoter and the CaMKIIα promoter (34, 35). However, it cannot be assumed that the WPRE enhances gene expression regardless of promoter used. Investigators should take this into consideration when designing vectors.

Another AAV technology that can be combined with systemic administration is a self-complementary AAV (scAAV) genome (36). Prior work with a ubiquitous promoter has shown that a greater number of cells are transduced using scAAV genome than single-stranded genome, however the presence of the WPRE in only the single-stranded genome due to packaging constraints may underlie these differences (3). Interestingly, two reports note a greater proportion of non-neuronal cells are transduced when using scAAV genomes packaged into PHP.eB (3, 37). Rincon et al. (37) report that using the hSyn1 promoter in a scAAV genome does not impact the neuron-specificity of the promoter. However, scAAV genomes have approximately half the payload capacity (approx. 2.4 kb) of single-stranded AAV.

One limitation of the present study is the analysis was restricted to 4 weeks after injection of the AAV. Therefore, it is not known if there is attenuation of expression with time. Jackson et al. (9) reported decreased GFP expression driven by hSyn1 in the cerebellum of rats 22 weeks post-injection. Investigators should take into consideration the length of transgene expression required when designing vectors.

Future studies will explore the possibility of using intravenous administration of AAV as a powerful tool to deliver potential therapeutics for neurodegenerative diseases, such as Alzheimer's disease, that impact disparate and widespread brain regions. Similarly, intravenous administration of AAV may allow developing an adult viral model of neurodegenerative disorders. Some germ line transgenic models of Alzheimer's disease do not recapitulate the robust pathology and neuron loss observed in humans (such as Tg 2,576 mice) (38). Alternatively, other models aggressively develop pathology and neuron loss at an early chronological age. Because the transgene is expressed throughout the lifespan, it is not possible to ascertain the contributions of aging processes upon the induction and progression of human disease.

For both therapeutic and model development approaches for neurodegeneration, restricting expression to neurons in the brain will be important to avoid off-target effects and limit potential confounds. Herein we demonstrate that both hSyn1 and CaMKIIα restrict expression to neural tissue and neurons compared to the CAG promoter, while yielding comparable transgene expression and distribution throughout the brain. Either neuronal promoter would be a candidate for preclinical testing of gene therapy or development of a viral model of neurodegeneration.

## Data Availability Statement

The datasets presented in this study can be found in online repositories. The names of the repository/repositories and accession numbers can be found below: https://www.ncbi.nlm.nih.gov/genbank/, M55301.1; https://www.ncbi.nlm.nih.gov/genbank/, NC_000084.7.

## Ethics Statement

The animal study was reviewed and approved by International Animal Care and Use Committee at Michigan State University.

## Author Contributions

DJF performed cloning experiments, purified AAV, injected the animals, performed IHC experiments, IMARIS analysis, and wrote the manuscript. IPN performed western blotting experiments, qPCR experiments, IHC experiments, and wrote the manuscript. DF-P performed qPCR experiments and wrote the manuscript. MRR performed IHC experiments and wrote the manuscript. KRN performed cloning experiments and wrote the manuscript. MNG contributed to mouse breeding and husbandry. DM and MNG contributed to the experimental design, interpretation of the data, and wrote the manuscript. All authors contributed to the article and approved the submitted version.

## Conflict of Interest

DM is on the speakers bureau for Biogen, a consultant with Abbvie, and receives research support from Hesperos. The remaining authors declare that the research was conducted in the absence of any commercial or financial relationships that could be construed as a potential conflict of interest.

## Publisher's Note

All claims expressed in this article are solely those of the authors and do not necessarily represent those of their affiliated organizations, or those of the publisher, the editors and the reviewers. Any product that may be evaluated in this article, or claim that may be made by its manufacturer, is not guaranteed or endorsed by the publisher.
