# Supplementary material for: Toward Development of Neuron Specific Transduction After Systemic Delivery of Viral Vectors
Source: Front Neurol. 2021 Aug 26;12:685802. doi: 10.3389/fneur.2021.685802 (PMC8426581; doi:10.3389/fneur.2021.685802)
Supplement: Supplementary file 1 [file Table_1.DOCX]

**Supplementary Table 1: Sequence of promoters used in present study.**

| Promoter | Sequence |
| --- | --- |
| CaMKIIα | cattatggccttaggtcacttcatctccatggggttcttcttctgattttctagaaaatgagatgggggtgcagagagcttcctcagtgacctgcccagggtcacatcagaaatgtcagagctagaacttgaactcagattactaatcttaaattccatgccttgggggcatgcaagtacgatatacagaaggagtgaactcattagggcagatgaccaatgagtttaggaaagaagagtccagggcagggtacatctacaccacccgcccagccctgggtgagtccagccacgttcacctcattatagttgcctctctccagtcctaccttgacgggaagcacaagcagaaactgggacaggagccccaggagaccaaatcttcatggtccctctgggaggatgggtggggagagctgtggcagaggcctcaggaggggccctgctgctcagtggtgacagataggggtgagaaagcagacagagtcattccgtcagcattctgggtctgtttggtacttcttctcacgataaggtggcggtgtgatatgcacaatggctaaaaagcagggagagctggaaagaaacaaggacagagacagaggccaagtcaaccagaccaattcccagaggaagcaaagaaaccattacagagactacaagggggaagggaaggagagatgaattagcttcccctgtaaaccttagaacccagctgttgccagggcaacggggcaatacctgtctcttcagaggagatgaagttgccagggtaactacatcctgtctttctcaaggaccatcccagaatgtggcacccactagccgttaccatagcaactgcctctttgccccacttaatcccatcccgtctgttaaaagggccctatagttggaggtgggggaggtaggaagagcgatgatcacttgtggactaagtttgttcgcatccccttctccaaccccctcagtacatcaccctgggggaacagggtccacttgctcctgggcccacacagtcctgcagtattgtgtatataaggccagggcaaagaggagcaggttttaaagtgaaaggcaggcaggtgttggggaggcagttaccggggcaacgggaacagggcgtttcggaggtggttgccatggggacctggatgctgacgaaggctcgcgaggctgtgagcagccacagtgccctgctcagaagccccaagctcgtcagtcaagccggttctccgtttgcactcaggagcacgggcaggcgagtggcccctagttctgggggcagc |
| hSyn1 | gagggccctgcgtatgagtgcaagtgggttttaggaccaggatgaggcggggtgggggtgcctacctgacgaccgaccccgacccactggacaagcacccaacccccattccccaaattgcgcatcccctatcagagagggggaggggaaacaggatgcggcgaggcgcgtgcgcactgccagcttcagcaccgcggacagtgccttcgcccccgcctggcggcgcgcgccaccgccgcctcagcactgaaggcgcgctgacgtcactcgccggtcccccgcaaactccccttcccggccaccttggtcgcgtccgcgccgccgccggcccagccggaccgcaccacgcgaggcgcgagataggggggcacgggcgcgaccatctgcgctgcggcgccggcgactcagcgctgcctcagtctgcggtgggcagcggaggagtcgtgtcgtgcctgagagcgcagtcgaga |
| CAG | ctagatctgaattcggtaccctagttattaatagtaatcaattacggggtcattagttcatagcccatatatggagttccgcgttacataacttacggtaaatggcccgcctggctgaccgcccaacgacccccgcccattgacgtcaataatgacgtatgttcccatagtaacgccaatagggactttccattgacgtcaatgggtggactatttacggtaaactgcccacttggcagtacatcaagtgtatcatatgccaagtacgccccctattgacgtcaatgacggtaaatggcccgcctggcattatgcccagtacatgaccttatgggactttcctacttggcagtacatctacgtattagtcatcgctattaccatggtcgaggtgagccccacgttctgcttcactctccccatctcccccccctccccacccccaattttgtatttatttattttttaattattttgtgcagcgatgggggcggggggggggggggggcgcgcgccaggcggggcggggcggggcgaggggcggggcggggcgaggcggagaggtgcggcggcagccaatcagagcggcgcgctccgaaagtttccttttatggcgaggcggcggcggcggcggccctataaaaagcgaagcgcgcggcgggcgggagtcgctgcgacgctgccttcgccccgtgccccgctccgccgccgcctcgcgccgcccgccccggctctgactgaccgcgttactcccacaggtgagcgggcgggacggcccttctcctccgggctgtaattagcgcttggtttaatgacggcttgtttcttttctgtggctgcgtgaaagccttgaggggctccgggagctagagcctctgctaaccatgttcatgccttcttctttttcctaca |
